# The Role of the Helper Lipid on the DNA Transfection Efficiency of Lipopolyplex Formulations

**DOI:** 10.1038/srep07107

**Published:** 2014-11-19

**Authors:** Zixiu Du, Mustafa M. Munye, Aristides D. Tagalakis, Maria D. I. Manunta, Stephen L. Hart

**Affiliations:** 1 Experimental and Personalised Medicines Section, UCL Institute of Child Health, 30 Guilford Street, London WC1N 1EH, UK

## Abstract

Multifunctional, lipopolyplex formulations comprising a mixture of cationic liposomes and cationic, receptor-targeting peptides have potential use in gene therapy applications. Lipopolyplex formulations described here are typically far more efficient transfection agents than binary lipoplex or polyplex formulations. It has been shown previously that the peptide component mediates both DNA packaging and targeting of the nanoparticle while in this report we investigate the contribution of the lipid component. We hypothesised that the lipid components synergise with the peptides in the transfection process by promoting endosomal escape after lipid bilayer fusion. Lipopolyplexes were prepared with cationic liposomes comprising DOTAP with either neutral lipid DOPE or DOPC. DOPE promotes fusogenic, inverted hexagonal lipid structures while DOPC promotes more stable laminar structures. Lipopolyplexes containing DOPE showed substantially higher transfection efficiency than those formulated with DOPC, both *in vitro* and *in vivo*. DOPE-containing lipopolyplexes showed rapid endosomal trafficking and nuclear accumulation of DNA while DOPC-containing formulations remained within the late endo-lysosomal compartments. These findings are consistent with previous finding for the role of DOPE in lipoplexes and support the hypothesis regarding the function of the lipid components in lipopolyplexes. These findings will help to inform future lipopolyplex design, strategies and clinical development processes.

Synthetic, non-viral vectors offer advantages over viral vectors for *in vivo* gene therapy in that they are less immunogenic, have fewer packaging constraints and are safer^1^,^2^. Cationic lipoplexes and polyplexes predominate in the non-viral vector field but increasingly lipopolyplex formulations, which are combinations of lipids with peptides or polymers, are being explored as appreciation develops of their wider range of functionalities and higher transfection efficiencies^3^,^4^,^5^,^6^,^7^,^8^,^9^,^10^,^11^. Further detailed functional and structural studies are required to understand the properties of lipopolyplexes, how to formulate components and to develop improved formulations.

We are developing a lipopolyplex formulation termed a Receptor Targeted Nanocomplex (RTN), which is a mixture of cationic, receptor-targeting peptides and cationic liposomes with plasmid DNA (pDNA)^12^,^13^,^14^,^15^,^16^,^17^. The lipid and peptide components of RTNs feature modular design elements that enable their functionality to be dissected at the molecular level^13^. We have shown previously that the peptide mediates DNA packaging and receptor targeting and so the focus of this study was to investigate the function of the lipids and how they contribute to the transfection efficiency of RTN lipopolyplexes. Endosomolysis is a major obstacle to transfection with peptide-DNA formulations and so we hypothesised that addition of the liposome to the peptide might enhance transfection by promoting fusion with the endosomal membrane, leading to improved cytoplasmic release of the DNA^13^,^18^. The neutral lipid DOPE in cationic lipoplex formulations enables higher transfection efficiencies as the conical structure of this lipid promotes the formation of inverted hexagonal structures that rapidly fuse with the endosomal lipid bilayer, independent of charge, thus enabling cytoplasmic release of the DNA. Substitution of DOPE for DOPC, a neutral lipid that favour more stable lamellar structures, greatly reduces lipoplex transfection efficiency^18^,^19^,^20^,^21^,^22^.

In this study, we aimed to explore whether the lipid components of the RTN lipopolyplex played a similar role in endosomal membrane fusion to that of lipoplexes and whether this contributed to their improved transfection efficiency. RTN formulations were therefore formulated with peptides mixed with cationic liposomes at the same charge density, containing either the fusogenic neutral lipid DOPE, or the non-fusogenic, neutral lipid DOPC. We hypothesised that if charge density of the liposome component was the more important factor then substituting the neutral lipid component would not affect transfection efficiency whereas if fusogenic properties were more important, the DOPE formulations would be superior to the DOPC formulations. The biophysical properties, transfection efficiencies and intracellular trafficking properties of each were investigated and compared to relate structural differences to functionality. Finally, *in vivo* transfection efficiencies of RTNs containing DOPE or DOPC were compared to assess the relevance of *in vitro* studies to *in vivo* applications.

This study will enable us to understand in more detail how such formulations function and to develop them further for gene therapy applications.

## Methods

The lipids (Supplementary Fig. 1) 1,2-dioleoyl-3-trimethylammonium-propane (DOTAP), 1,2-dioleoyl-sn-glycero-sn-3-phosphatidylcholine (DOPC), 1,2-dioleoyl-sn-glycero-3-phosphatidyl-ethanolamine (DOPE) and Rhodamine-DOPE were purchased from Avanti Polar Lipids (Birmingham, Alabama, USA). The peptide sequences K_16_GACSERSMNFCG (K_16_E) and K_16_GACYGLPHKFCG (K_16_Y) were purchased from China Peptides (Shanghai, China) and dissolved to 10 mg/mL in DNase/RNase free water (Invitrogen, Paisley, UK). The plasmid pCI-Luc comprised the luciferase gene of pGL3 (Invitrogen, Paisley, UK) subcloned into the eukaryotic expression vector pCI (Promega, Southampton, UK).

### Liposome, lipoplex and lipopolyplex formulation

The liposomes L_C1_, L_C2_ and L_C3_ comprised formulations of DOTAP: and DOPC weight ratios of 0.5:1, 1:1 and 3:1 respectively (DOTAP:DOPC). The homologous DOTAP/DOPE liposomes L_E1_, L_E2_ and L_E3_ comprised a mixture of DOTAP and DOPE at weigh ratios of 0.5:1, 1:1 and 3:1 respectively. Liposomes were prepared by mixing the component lipids dissolved in chloroform to a final volume of 200 μL, followed by rotary evaporation in a Buchi Rotavapor (Oldham, UK) under a partial vacuum to produce a thin lipid film. Lipids were then rehydrated with 1 ml of distilled water whilst rotating overnight and then sonicated in a XB3 Ultrasonic Bath (Grant Instruments, Cambridge, UK) until clear to make the liposome solution at 1 mg/ml.

Lipoplexes (LD) were then formed by mixing the cationic liposomes with pDNA at a weight ratio of 4:1 lipid (L):DNA (D), in distilled water or OptiMEM (Invitrogen, Paisley, UK) with DNA at a final concentration of 1 μg per 100 μl. LD mixtures were then incubated for 30 min at room temperature to stabilise before use. Lipid (L):Peptide (P):DNA (D) formulations were prepared by mixing the different liposomes with peptide in the order L:P:D at weight ratios of 0.5:4:1, 0.75:4:1, 1:4:1 and 2:4:1.

### Hydrodynamic size and zeta potential measurements

The hydrodynamic size and zeta potential of the liposomes (5 μg lipid per ml), LD and LPD nanocomplexes (both at 2 μg/ml with respect to plasmid DNA) in distilled water were determined by dynamic light scattering (DLS) and laser Doppler velocimetry, respectively, using a Malvern Nano ZS (Malvern Instruments, Malvern, UK). The z-average data are reported with Polydispersity Index (PDI) values of less than 0.3 accepted as representing a monodisperse population of particles. The overall charge (zeta potential) that the nanocomplex acquires in water while attracted by the oppositely charged electrode was measured and reported as strength field unit (mV).

### Negative staining transmission electron microscopy (TEM)

A 5 μl aliquot of LPD nanocomplexes was applied onto a 300-mesh copper grid coated with a Formvar/carbon support film (Agar Scientific, Essex, UK) then, after a few seconds, dried by blotting with filter paper. The sample was then negatively stained with 1% uranyl acetate for a few seconds, before blotting with filter paper and air dried. Imaging was carried out with a Philips CM120 BioTwin Transmission Electron Microscope and operated at an accelerating voltage of 120 kV.

### Cell culture and luciferase transfection

The human bronchial epithelial cell line 16HBE14o- (D. Gruenert, San Francisco) was maintained in Eagle's Minimal Essential Medium (MEM; Sigma-Aldrich, Dorset, UK) supplemented with 10% (v/v) foetal bovine serum (FBS), 100 U/mL penicillin, 100 mg/mL streptomycin, and 2 mmol/l L-glutamine. The murine neuroblastoma cell line Neuro-2A (ATCC, Manassas, VA, USA) was cultured in Dulbecco's Modified Eagle Medium (DMEM), 1% (v/v) non-essential amino acids, 1 mM sodium pyruvate and 10% (v/v) FBS (Invitrogen, Paisley, UK). Both cell lines were incubated at 37°C in a humidified atmosphere with 5% CO_2_. For transfections, cells were seeded into 96-well plates at 2.5 × 10^4^ cells per well then LD or LPD nanocomplexes in OptiMEM added to each well containing 250 ng of plasmid DNA in 200 μl in replicates of six. Plates were centrifuged at 483 × *g* for 5 min to promote sedimentation of the nanocomplexes and incubated for a further 24 h. Cells were then lysed with Reporter Lysis Buffer and a chemiluminescence assay was performed to measure luciferase activity (Promega, Southampton, UK). Protein concentration in the lysate was determined using a Bio-Rad protein assay (Hemel Hempstead, UK) then luciferase activity expressed as relative light units (RLU) per milligram of protein.

### Confocal microscopy

Fluorescently-labelled LPD formulations were prepared with L_C2_ or L_E2_ liposomes containing Rhodamine-DOPE at 0.5% of total lipid while 50% of the pCI-Luc was labelled with Cy5 dye using the Universal Linkage System Nucleic Acid Labelling Kit (Kreatech Diagnostics, Amsterdam, Netherlands). 15 × 10^4^ 16HBE14o- cells were seeded onto glass coverslips in 6-well plates and incubated for 24 h at 37°C in a humidified atmosphere with 5% CO_2_. Fluorescently-labelled nanocomplexes L_C2_PD-0.75 and L_E2_PD-0.75 (Table 1) were added to the cells (1.5 μg DNA/well), the plates were centrifuged at 483 × g for 5 min and then incubated for 6 h or 22 h. The cells were then fixed with 4% paraformaldehyde, permeabilised with 0.1% Triton in phosphate-buffered saline, blocked with 3% bovine serum albumin (BSA), and stained for 2 h at room temperature with anti-Lysosomal-associated membrane protein 1 (LAMP-1) primary antibody (1:250 dilution in 1% BSA; product number H4A3, Abcam, Cambridge, UK), washed with phosphate-buffered saline and then incubated for 1 h at room temperature with Alexafluor 488 goat anti-mouse secondary antibody (1:1000 dilution in 1% BSA, Invitrogen, Paisley, UK) and DAPI (0.1 mg mL^−1^, Sigma-Aldrich, Poole, UK). The cells were washed and sealed in mounting media (Invitrogen, Paisley, UK) before visualising with a 63 × oil immersion objective (N.A. 1.4) under a Carl Zeiss LSM710 laser scanning microscope system (Carl Zeiss, Jena, Germany).

### In vivo delivery and luciferase assay on lung tissues

Female CD1 mice were purchased from Charles River (Margate, UK). All procedures were approved by UCL animal care policies and were carried out under Home Office Licenses issued in accordance with the United Kingdom Animals (Scientific Procedures) Act 1986 (UK). L_C1_PD and L_E1_PD complexes were prepared at a weight ratio of L:P:D of 0.75:4:1 essentially as described previously^15^ at a final pDNA concentration of 0.33 mg/ml. 4-week old female CD1 mice were instilled oropharyngeally with nanocomplexes in 55 μl water containing 18.15 μg of pCI-Luc, with untreated mice used as controls (n = 3). 24 h following instillation, the mice were culled and their lungs extracted and snap frozen. Lungs were defrosted on ice, submerged in reporter gene assay lysis buffer (Roche, Basel, Switzerland), homogenized with a Precellys24 tissue homogenizer (Stretton Scientific, Stretton, Derbyshire, UK) and then centrifuged at 14,170 × *g* for 10 min at 4°C. The supernatant was removed and centrifuged for a further 10 min at 4°C then used in luciferase assays. Results were expressed as relative luminescence units per milligram of protein (RLU/mg).

### Statistical analysis

Data are shown as mean ± standard deviation (S.D.). Statistical analysis was performed by unpaired t-test using GraphPad Prism Statistics software version 5.01 (GraphPad Software, Inc, La Jolla, CA, USA). A difference was considered statistically significant when *P* < 0.05.

## Results

### The morphology of the representative samples

**Figure 1:**
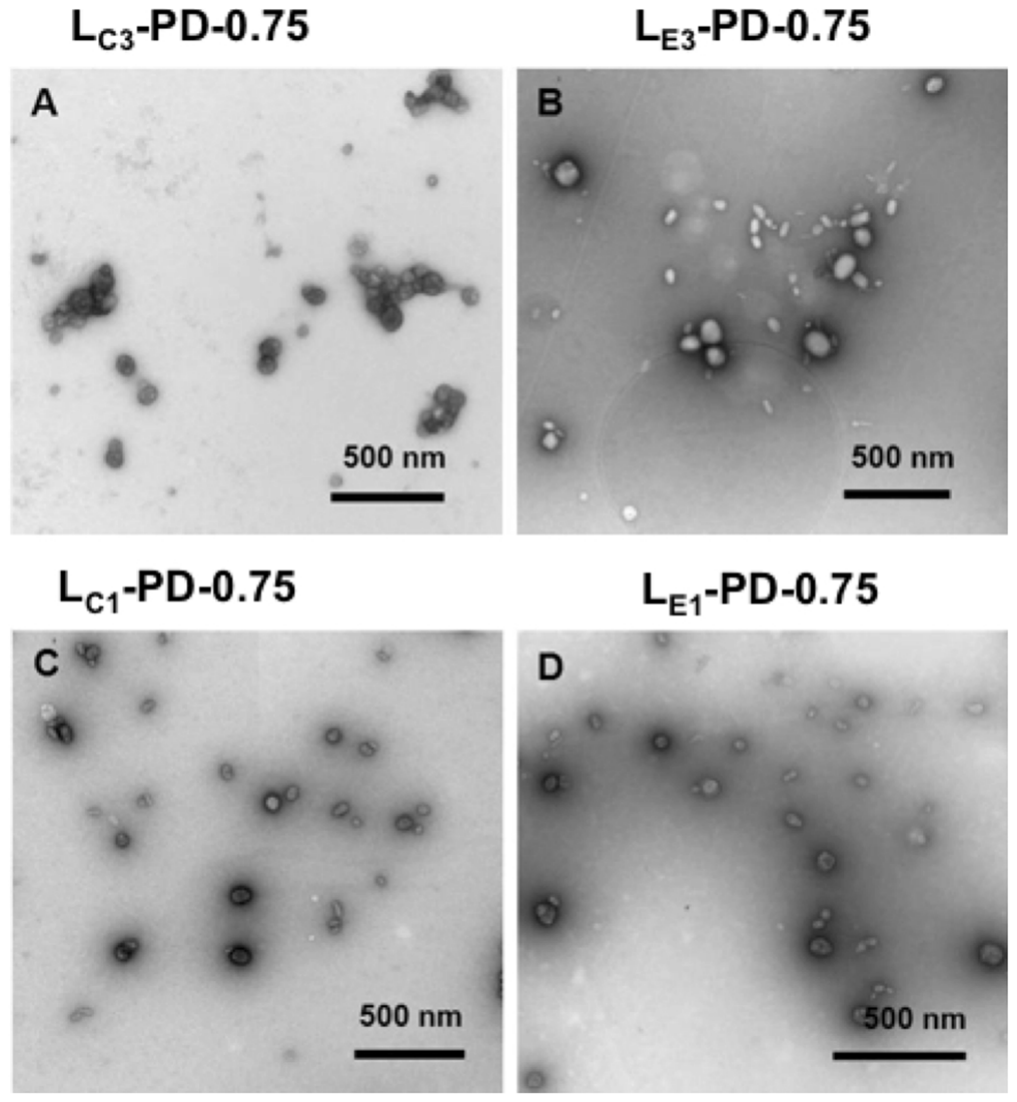
Transmission electron microscopy of LPD nanocomplexes. (A) Images of L_C1_PD-0.75, (B) L_E1_PD-0.75, (C) L_C3_PD-0.75 and (D) L_E3_PD-0.75 nanocomplexes. Positively-stained spherical particles and negatively stained rods are evident in all images. Scale bar is 500 nm for all images.

Biophysical analysis by transmission electron microscopy (TEM) (Fig. 1) and dynamic light scattering analysis (Table 1) showed that L_C1_PD-0.75 formulations formed spherical particles of 78 ± 0.6 nm (PDI < 0.3) and a zeta potential of 36.6 ± 1.8 mV with a tendency to agglomerate (Fig. 1A). L_E1_PD-0.75 formulations, in which DOPE replaced DOPC, formed mostly spheres (Fig. 1B) of a similar size 85.5 ± 0.6 nm (PDI < 0.3) but much higher zeta potential at +62.5 mV than L_C1_PD-0.75. On the other hand, L_C3_PD-0.75 and L_E3_PD-0.75 both formed mostly spheres of both similar size about 74 nm (PDIs < 0.3) and zeta potential at +42 and +39 mV respectively. Both also formed some rod-like structures in addition to sphere at about 10 nm wide and 40 nm long (Fig. 1C, D). Increasing the DOTAP content in the formulations L_C1_PD-0.75 (78 nm) to L_C2_PD-0.75 (68 nm) to L_C3_PD-0.75 (74 nm) produced no pattern in size changes but for the homologous DOPE formulations size decreased from 86 nm to 74 nm.

### Luciferase transfection of LD and LPD nanocomplexes

The transfection efficiency of L_C1-3_D complexes (4:1 L:D weight ratios) improved with decreasing Φ_DOPC_ in both 16HBE14o- and Neuro-2A cells (Supplementary Fig. 1), while there was no consistent trend when varying Φ_DOPE_ for the transfection efficiency of L_E1-3_D lipoplexes. This pattern of results was consistent with previously reported transfection data with DOPC and DOPE-containing lipoplexes^19^,^20^.

**Figure 2:**
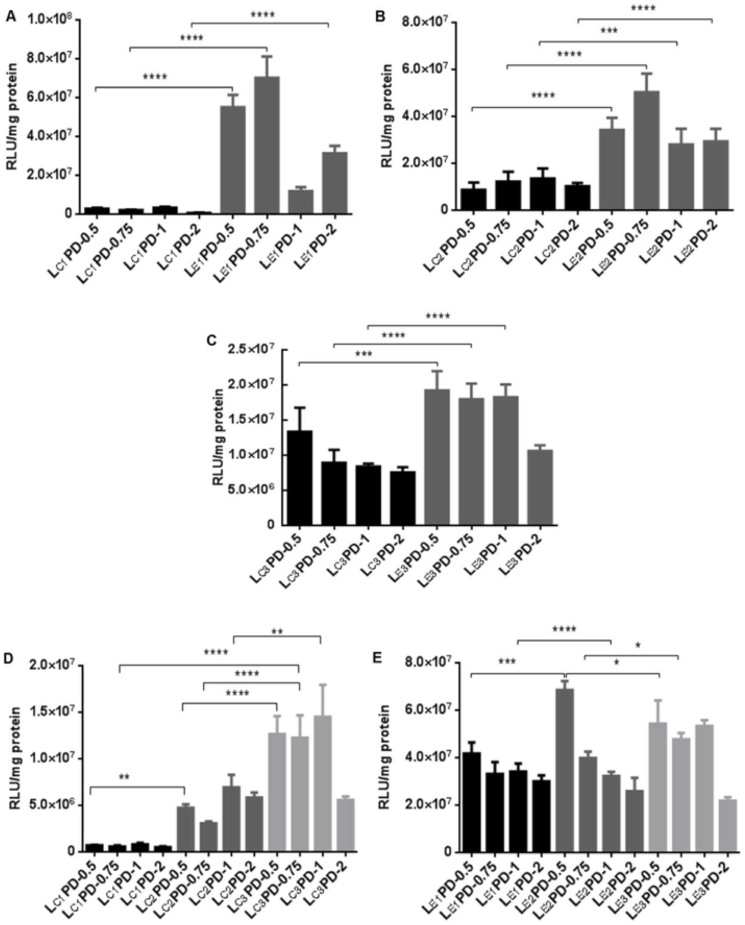
Transfection efficiencies in 16HBE14o- cells of lipopolyplexes formulated with the targeting peptide K_16_GACSERSMNFCG, plasmid pCl-Luc and liposomes with DOPE or DOPC at different weight fractions. LPD lipopolyplexes were formulated at weight ratios of liposomes:DNA of 0.5, 0.75, 1 or 2, as shown in labels. Lipopolyplexes were compared containing lipids L_C1_ and L_E1_ (A), L_C2_ and L_E2_ (B), L_C3_ and L_E3_ (C), L_C1-3_ (D) and L_E1-3_ (E). Transfection efficiency was measured by luciferase activity and expressed as relative light units per mg of protein (RLU/mg). Values are the means of 6 replicates ± standard deviation. Four stars represent p < 0.0001 and three stars represent p < 0.001.

The transfection efficiency of L_E_PD was significantly higher than that of L_C_PD nanocomplexes at the same weight ratio of liposome to pDNA (Fig. 2A–C). The transfection efficiencies of L_C_PD lipopolyplexes increased with decreasing Φ_DOPC_ (Fig. 2D) while there was no consistent trend seen with decreasing Φ_DOPE_ (Fig. 2E).

### Confocal microscopy

**Figure 3:**
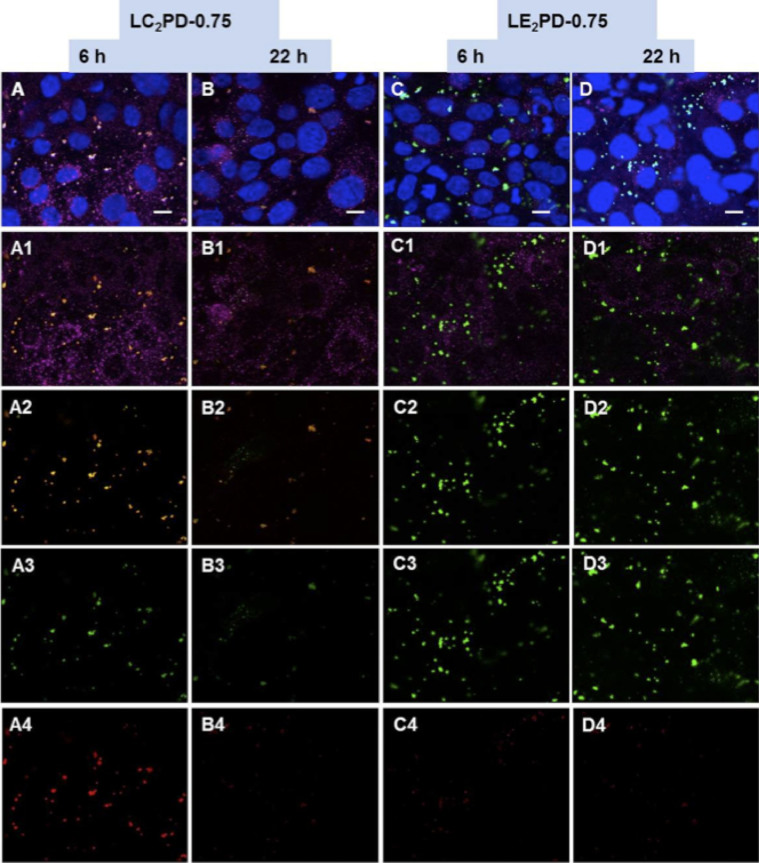
Confocal microscopy images of the intracellular localisation of lipopolyplexes identified by Cy5-DNA and rhodamine-labelled liposome. 16HBE14o- cells were transfected for 6 h or 22 h with the nanocomplexes. Representative images of lipopolyplexes are shown: L_C2_PD-0.75 at 6 h (A), L_C2_PD-0.75 at 22 h (B), L_E2_PD-0.75 at 6 h (C), L_E2_PD-0.75 at 22 h (D). Panels A–D represent merged images showing Rhodamine-DOPE liposomes (red) and Cy5-labelled-DNA (green) taken up by 16HBE14o- epithelial cells. Inside the cells, the late endosomal/lysosomal compartments are identified by lysosomal associated membrane protein 1 (LAMP-1; magenta) and the nuclei by DAPI (blue). Panels A1–D1 show the localisation of Rhodamine-DOPE lipid and Cy5-labelled-DNA relative to LAMP-1; panels A2–D2 show Rhodamine-labelled lipid and Cy5-labelled-DNA; A3–D3 indicate Cy5-labelled-DNA alone, whereas panels A4–D4 show rhodamine-labelled liposome only (Scale bar = 10 μm for all images).

Cy-5-labelled pDNA (green) and Rhodamine-labelled liposome (red) were used to monitor the trafficking of LPD inside 16HBE14o- cells. Subcellular localisation of the vector components was assessed in relation to the late endosomal-lysosomal compartment marker (LAMP-1, magenta) and DAPI- stained nuclei (blue). At 6h, L_C2_PD-0.75 accumulated in perinuclear lysosomes (yellow dots representing colocalised Cy5-DNA and rhodamine-lipids) (Fig. 3A) and even after 22 h, most pDNA still resided in the late endosomes/lysosomes with very little free pDNA found in the cytoplasm or the nucleus (Fig. 3B). In contrast, free pDNA from L_E2_PD-0.75 complexes was found in the cytoplasm and nucleus as early as 6 h (Fig. 3C) which further increased by 22 h (Fig. 3D).

### Luciferase assay of lung extracts

**Figure 4:**
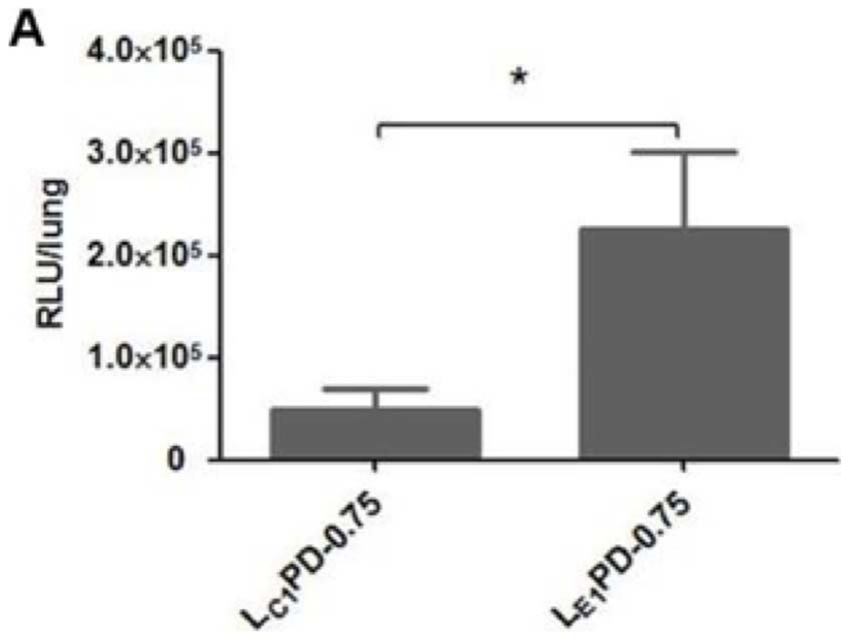
In vivo transfections of mice lungs. Luciferase expression in mice lungs was detected 24 h after oropharyngeal instillation of L_C1_PD-0.75 or L_E1_PD-0.75 nanocomplexes.

Finally we determined whether the *in vitro* results translated to an *in vivo* application by delivery to the airways of murine lungs. Twenty-four hours after administration, luciferase assays were performed on lung extracts of mice treated with either L_C1_PD-0.75 or L_E1_PD-0.75 nanocomplexes. These formulations were selected as they exemplified the effects of the neutral lipids on transfection efficiency *in vitro*. The luciferase expression from L_E1_PD-0.75 was five-fold higher than L_C1_PD-0.75 (Fig. 4) while the same formulations transfecting 16HBE14o- cells confirmed that L_E1_PD-0.75 was again significantly better than the L_C1_PD-0.75 nanocomplexes (Supplementary Figure 2).

## Discussion

We are developing formulations of lipids with peptides and DNA which self-assemble into nanoparticles electrostatically on mixing at optimised ratios of components^13^,^14^,^15^,^16^,^17^. The transfection efficiency of these lipopolyplexes is substantially higher than either the lipoplex or polyplex containing the same liposomes or peptides and so we aim to clarify the mechanism of synergy of the lipid and peptide components. We hypothesised that, while the peptide mediates packaging and targeting^15^,^16^,^21^ lipid components of lipopolyplexes may aid in endosomal disruption and the trafficking of the nucleic acid into the cytoplasm and therefore reasoned that strongly fusogenic lipids, such as DOPE, should display higher transfection efficiencies in lipopolyplexes than DOPC that promotes more stable laminar lipid bilayer structures.

Biophysical analysis revealed that lipopolyplex formulations containing DOPE and DOPC lipids in variable proportions, were of a consistent size and charge with only marginal affects of alterations to the lipid composition of each formulation. TEM images showed that there were no significant differences in lipopolyplex morphology with a mixture of spheres and rods observed in most cases of similar sizes. Despite only minor differences in biophysical properties, transfection results of L_C_PD and L_E_PD lipopolyplexes revealed that at higher weight fractions of neutral lipids (i.e., those containing L_E1_ and L_C1_) the transfection efficiency of L_E_PD formulations was significantly higher than that of L_C_PDs. As the weight fraction of neutral lipids decreased and the liposome charge density increased, the relative transfection efficiency of L_C_PDs was enhanced although even with lipids at the highest charge density, i.e., L_E3_ and L_C3_, the transfection efficiency of L_E_PD formulations remained approximately twice that of L_C_PD formulations. These same trends were also observed with L_E_D and L_C_D lipoplex transfections, with L_C_D transfection efficiencies increasing significantly with higher charge density while the L_E_D formulations showed no consistent trend in relation to charge density. The transfection enhancement of LD and LPD complexes containing DOPE rather than DOPC indicates the importance of the inverted hexagonal structure of the DOPE lipid in enabling endosomal membrane fusion and release of DNA compared to the lamellar DOPC structure. Cellular trafficking studies confirmed that the DOPC-containing cationic liposomes showed limited ability to escape from the endosome leading to lower transfection efficiencies of lipopolyplexes.

Finally, we investigated whether DOPE can improve the transfection efficiency of the RTN lipopolyplex *in vivo* by delivery to murine lungs (Fig. 4). L_E1_PD-0.75 yielded luciferase reporter gene expression levels five times higher than that of L_C1_PD-0.75 formulations. Thus we have shown that the advantages of DOPE for *in vitro* transfections in lipopolyplexes translated to an *in vivo* system. This result contrasted with a previous comparative study of DOPE-containing lipopolyplexes where the formulation was administered systemically and poor transfection efficiency was observed. However, this difference was due to serum interactions with lipopolyplexes neutralising DOPE activity before actually entering cells^22^.

We have shown that the fusogenic properties of DOPE, as in lipoplex formulations, are essential for the transfection function of lipopolyplex formulations but we have shown additionally that this property can be combined with the targeting and packaging properties of the peptide component to produce a significant enhancement of transfection efficiency compared to the lipoplex alone. The cationic lipid is important for the stability of the liposome component and its electrostatic association with DNA in formulating the lipopolyplex but probably does not contribute to membrane interactions in the cellular transfection pathway. These findings will be important in designing improved lipopolyplex formulations.

## Author Contributions

Z.D. performed most of the experimental work, drafted the manuscript and prepared the figures. M.Munye provided advice and input into transfection protocols for Figures 2 and 4 and for biophysical measurements in Table 1. A.D.T. assisted in producing the data for Figure 1 and 4. M.Manunta assisted with confocal studies in Figure 3. S.H. supervised all aspects of the work and produced the final manuscript. All authors read and approved the final manuscript.

## Supplementary Material

Supplementary Information Supplementary Information

## Figures and Tables

**Table 1:** Hydrodynamic size and zeta potential of LPD nanocomplexes formed at different weight ratios L_C1_:P:D and L_E1_:P:D as measured by dynamic light scattering (n = 3, mean ± standard deviation)

Formulation (LPD)	L:P:D (weight%)	Size (nm)	PDI	Zeta (mV)
L_C1_PD-0.5	0.5:4:1	65.9 ± 1.1	0.145	33.8 ± 0.4
L_C1_PD-0.75	0.75:4:1	77.9 ± 0.6	0.144	36.6 ± 1.8
L_C1_PD-1	1:4:1	68.7 ± 0.5	0.153	40.6 ± 2.8
L_C1_PD-2	2:4:1	76.1 ± 0.1	0.247	60.7 ± 2.7
L_E1_PD-0.5	0.5:4:1	79.4 ± 0.3	0.245	68.8 ± 11.2
L_E1_PD-0.75	0.75:4:1	85.5 ± 0.6	0.299	62.5 ± 1.0
L_E1_PD-1	1:4:1	100.6 ± 0.9	0.333	64.6 ± 2.0
L_E1_PD-2	2:4:1	120.5 ± 0.8	0.445	65.1 ± 0.7
L_C2_PD-0.5	0.5:4:1	84.8 ± 0.4	0.202	44.4 ± 0.4
L_C2_PD-0.75	0.75:4:1	68.4 ± 0.5	0.196	56.5 ± 0.5
L_C2_PD-1	1:4:1	85.2 ± 1.4	0.155	51.3 ± 1.4
L_C2_PD-2	2:4:1	118.3 ± 1.1	0.237	45.5 ± 1.2
L_E2_PD-0.5	0.5:4:1	65.7 ± 0.4	0.240	58.9 ± 1.5
L_E2_PD-0.75	0.75:4:1	92.1 ± 1.3	0.371	57.7 ± 2.7
L_E2_PD-1	1:4:1	83.7 ± 0.3	0.315	63.3 ± 1.2
L_E2_PD-2	2:4:1	94.6 ± 2.0	0.442	67.7 ± 1.2
L_C3_PD-0.5	0.5:4:1	76.1 ± 1.9	0.288	40.5 ± 1.5
L_C3_PD-0.75	0.75:4:1	74.5 ± 0.7	0.158	41.8 ± 1.7
L_C3_PD-1	1:4:1	67.6 ± 0.3	0.250	46.8 ± 4.7
L_C3_PD-2	2:4:1	99.6 ± 1.7	0.350	56.0 ± 1.6
L_E3_PD-0.5	0.5:4:1	78.0 ± 0.6	0.263	53.4 ± 8.1
L_E3_PD-0.75	0.75:4:1	73.5 ± 0.9	0.218	38.5 ± 2.5
L_E3_PD-1	1:4:1	70.1 ± 1.0	0.194	41.3 ± 2.2
L_E3_PD-2	2:4:1	70.4 ± 0.5	0.195	48.5 ± 1.9
