# Supplementary material for: The Role of the Helper Lipid on the DNA Transfection Efficiency of Lipopolyplex Formulations
Source: Sci Rep. 2014 Nov 19;4:7107. doi: 10.1038/srep07107 (PMC4236742; doi:10.1038/srep07107)
Supplement: Supplementary Information [file srep07107-s1.doc]

**The Role of the Helper Lipid on the DNA Transfection Efficiency of Lipopolyplex Formulations**

Zixiu Du^a^,, Mustafa M. Munye, Aristides D. Tagalakis, Maria D.I. Manunta, Stephen L. Hart^b^

Molecular Immunology Unit, UCL Institute of Child Health, 30 Guilford Street, London WC1N 1EH, UK

^a^ Current address: School of Pharmacy, Shanghai Jiao Tong University, 800 Dongchuan Road, Shanghai 200240, China

^b^ Author for communication

Communicating author:

Professor Stephen L Hart

Molecular Immunology Unit,

UCL Institute of Child Health,

30 Guilford Street,

London

**Supplementary Table 1** Hydrodynamic size and zeta potential of LD lipoplexes formed at 4:1 weight ratios of L_C_:D or L_E_:D as measured by dynamic light scattering (n = 3, mean ± standard deviation).

| Lipoplexes | L:D (w %) | Size (nm) | **^a^**PDI | Zeta (mV) |
| --- | --- | --- | --- | --- |
| L_C1_D | 4:1 | 121.8 ± 1.2 | 0.094 | -26.9 ± 0.4 |
| L_C2_D | 4:1 | 142 ± 1.5 | 0.136 | -7.7 ± 1.9 |
| L_C3_D | 4:1 | 267.3 ± 2.9 | 0.342 | 37.5 ± 8.2 |
| L_E1_D | 4:1 | 524 ± 52.4 | 0.518 | -29.1 ± 0.4 |
| L_E2_D | 4:1 | 545 ± 2.0 | 0.174 | 35.8 ± 0.1 |
| L_E3_D | 4:1 | 126.6 ± 2.2 | 0.087 | 46.2 ± 4.6 |

^a^ represents the weight ratio of lipids to pDNA.

^b^ represents the size PDI of lipoplexes.

**A**

**B**


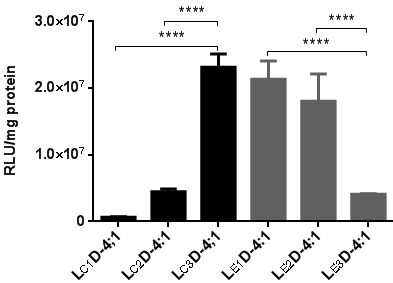

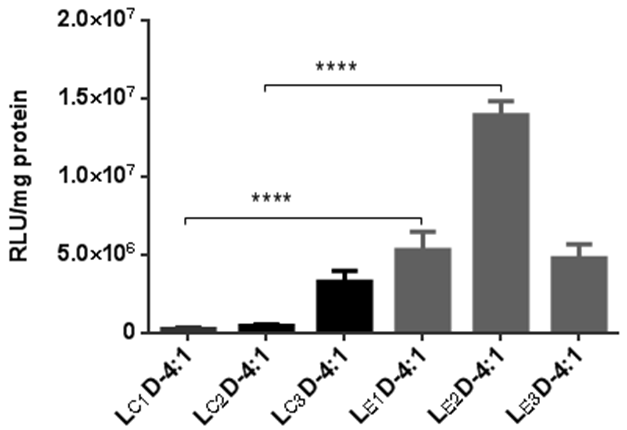


**Supplementary Figure 1.** Transfection efficiencies of LD lipoplexes in 16HBE14o- (A) and Neuro-2A cells (B). Luciferase reporter gene activity was expressed as relative light units per mg of protein (RLU/mg). Values of a representative experiment are the means of 6 replicates ± standard deviation. Four stars represent p < 0.0001.

**
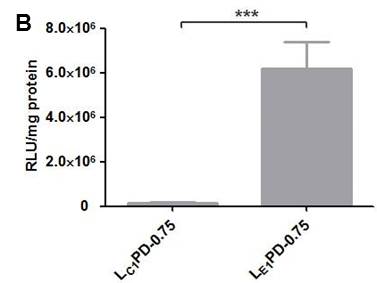
**

**Supplementary Figure 2.** The transfection efficiencies of the RTNs used for in vivo experiments were assessed *in vitro* in 16HBE14o- cells.
